# Borderline in a linear city: Urban living brings borderline personality disorder to crisis through neuroplasticity—an urgent call to action

**DOI:** 10.3389/fpsyt.2024.1524531

**Published:** 2025-01-06

**Authors:** Mohamed Hesham Khalil

**Affiliations:** Department of Architecture, University of Cambridge, Cambridge, United Kingdom

**Keywords:** mental health, environmental enrichment, borderline personality disorder, stress, neuroscience, built environment, natural environment, public health

## 1 Introduction

Since its identification in the late 1930s by the American psychiatrist and psychoanalysis Adolph Stem (1), there has been a steeper increase in borderline personality disorder (BPD), specifically over the past few years (2). BPD is found in around 1.7% of the general population but in 15-28% of patients in psychiatric clinics or hospitals and in a large proportion of individuals seeking help for psychological problems as reported in general health facilities (3). BPD is one of the most complex disorders to date characterised by sudden shifts in identity, interpersonal relationships, impulsive behaviour, periodic intense anger, the feeling of emptiness, dissociative symptoms, and sometimes suicidal thoughts or behaviours (4). BPD results in severely poor outcomes from low occupational and educational attainment to disturbed and unsustainable relationships (5). BPD can be genetic, but the risk of developing BPD can also be environmentally-influenced, which this article shows that it may not only be due to an unhealthy social environment as commonly perceived, but also due to the physical environment to a great extent.

The critical problem is that BPD is often untreatable, which urges a critical exploration of the silent physical environment variables triggering BPD and pushing it to a critical risky crisis. Leichsenring et al. (6) have recently explained that up to 96% of patients with BPD who seek treatment receive at least one psychotropic drug (7), and 19% of BPD patients report taking four or more psychotropic drugs (8). No class of psychoactive medications has been proven to be consistently effective, and no medication has been proven for that (9). Leichsenring et al. (6) further explained that pharmacotherapy is not recommended for the treatment of any core symptom of BPD but only addresses discrete and severe depression, anxiety or psychotic symptoms for the shortest possible time and in crises. The complexity of the BPD symptoms and of effective psychoactive medication raises a critical need to understand this complexity at the brain level, specifically since the human brain is neuroplastic and continuously adapts to its environment. This is in line with current research efforts, where neuroscience is interested in understanding the connection between the physical environment and mental health (10) and exploring how the physical environment promotes sustainable adaptive neuroplasticity for mental health (11). However, to date, researchers only focus on the social environment to understand the gene-environment influences on BPD (12–14), but a plethora of evidence, presented shortly in this paper, suggests that the physical environment, particularly urbanism, can be the silent trigger and chronic worsener for BPD patients or subjects with genetic predisposition. By the early 20th century, when BPD was formally identified, cities, particularly in the United States and England, were undergoing rapid expansion. This may make it unsurprising that the evidence presented in this article explains the identification of BPD at the same time and in the same region.

This article adopts a novel perspective to prevent triggering BPD in populations with a genetic predisposition, facilitate the treatment of current BPD patients, reduce suicide, and show how the physical environment can benefit the general population, who may not be as vulnerable as BPD patients or genetically predisposed individuals.

## 2 Method

A comprehensive search for existing reviews was conducted using the Scopus database with no specific timeframe ranges or subject area limits. Firstly, the search was conducted using the keywords ((“borderline personality disorder”) AND (amygdala OR hippocampus OR cortex)). This is because, in many people with BPD, those three brain regions (amygdala, hippocampus, orbitofrontal cortex) are either smaller than expected or have unusual activity levels. The amygdala is responsible for emotional regulation, the hippocampus for behavioral regulation and self-control, and the orbitofrontal cortex for planning and decision-making (15). The BPD symptoms introduced earlier are strongly associated with the functions of those affected brain regions. Secondly, due to the rising interest in promoting neurosustainability through the physical environment by focusing on the same three brain regions (amygdala, hippocampus, cortex) (11), further research was conducted using Scopus on the impact of both built environment and natural environment on the three identified brain regions to observe any potential association with the maladaptive plasticity outcomes observed in people with BPD in the first search through Scopus. The search primarily focuses on studies conducting brain scans, while supplementary studies using EEG are searched for and introduced when combined with MRI or relevant to enriching the discourse. The following sections discuss BPD in the brain, urbanicity and maladaptive plasticity outcomes related to maladaptive plasticity in people with BPD, nature-driven neurosustainability for adaptive neural responses and potentially reduced symptoms, and last but not least, presenting final thoughts with potential implications and limitations at the urban, architectural and interior scales.

## 3 Maladaptive neuroplasticity of brains with BPD

The body of literature on the intersection of neuroscience with BPD is extensive, beginning in the early twenty-first century, but despite the strong identification of the key brain regions with maladaptive neuroplasticity, the main BPD problem remains unresolved, and more BPD patients are not responding to therapy.

In the first decade of the millennium, several neurological studies emerged that were reviewed by multiple researchers who helped understand the neurological underpinnings of BPD. New and Siever (16) discussed how impulsive aggression in BPD involves the lack of the prefrontal orbital cortex exerting an inhibitory control over aggressive behaviors in normal individuals. Teicher et al. (17) show that severe early stress is one of the factors having the potential to alter brain development, potentially leading to attenuated development of the left neocortex, hippocampus and amygdala that all increase the risk of developing several mental health problems, including but not limited to BPD. Saper and Lake, (18) reported that women patients with BPD have reduced hippocampal volume along with hypermetabolism in the premotor, prefrontal, and anterior cingulate cortex. Sala et al. (19) showed in their review that BPD may suffer hippocampal atrophy as a result of stressors. McCloskey et al. (20) also confirmed in their review that BPD is associated with a decrease in hippocampal volume. However, they also showed that BPD is associated with increased amygdala activation of general (though not personal) emotional stimuli, along with prefrontal hypermetabolism and functional dysregulation. Geuze et al. (21) also confirmed in their review the relationship between BPD and reduced hippocampal volume. Brendel et al. (22) have reviewed the evidence for dysfunction in certain frontolimbic regions that lead to a mechanistic model of symptom formation in BPD, proposing that future neuroimaging studies of BPD should encompass multilevel observations including not only the structural but also functional dynamics among others. On the contrary, Ivanovski and Malhi (23) have shown that mindfulness-based therapeutic interventions were evidenced using fMRI and EEG studies to be effective in the treatment of multiple psychiatric conditions, including but not limited to BPD. After previously discussing the amygdala-prefrontal disconnection in BPD New et al. (24, 25) followed up to show that the promising findings in BPD suggest that a diminished top-down control of affective responses is probably associated with the decreased responsiveness of midline regions of the prefrontal cortex that underlie the affective hyperresponsiveness in BPD. A meta-analysis confirmed the smaller volume found in both the right and left hippocampus and amygdala of BPD patients (26). Zimmerman and Choi-Kain (27) questioned whether the hypothalamic-pituitary-adrenal (HPA) axis functions normally in BPD because the activation of the HPA axis occurs to coordinate both behavioral and physiological responses to stress, showing through their review the possibility of developing an HPA-axis dysfunction in BPD.

The last decade continued to build up on neuro evidence that existed by that time, beginning with a clearer synthesis (28), and confirming earlier findings (29) before further research started exploring the neural underpinnings in more detail. The beginning is with a review by Wingenfeld et al. (30), who discussed that although stress seems to be associated with the development of BPD, the function of the HPA axis in BPD needed to be explored further, discussing it along with the hippocampus and amygdala and cortex patterns discussed previously. They hypothesised that BPD is characterised by a dysfunctional regulation of the HPA axis, disturbances of the serotonergic system, and the identified maladaptive patterns of the hippocampus, amygdala and cortex. A meta-analysis was published a couple of years later by Ruocco et al. (31), aiming to evaluate whether the magnitude of the hippocampus and amygdala volume decreases and their associations with BPD, showing through 11 MRI-based studies that BPD patients showed an average of 11% and 13% decrease in the hippocampus and amygdala sizes, respectively, which is the minimum compared to earlier reports that it can even be up to 24% lower in the hippocampus volume and 20% in the amygdala volume (32). To explain before proceeding further, hippocampal volume and cell number reduced 20% - 35% in patients with depression (33), which explains the depression symptoms in BPD. Another meta-analysis by de-Almeida et al. (34) explained that the amygdalar volumes are reduced in patients with BPD, and this is confirmed in BPD patients without posttraumatic stress disorder (PTSD) but not in BPD patients with PTSD, concluding that comorbid PTSD cannot explain the reduced amygdalar volume in BPD patients. Besides the reduced hippocampal and amygdala volumes in patients with BPD, O’Neill and Frodl (35) showed in their review an exaggerated amygdala activity in BPD patients when confronted with emotion-related stimuli. Mak and Lam (36) showed in their review that EEG studies explain the right hemispheric deficit in high-order cortical inhibition in patients with BPD while confirming that the brain structures implicated in BPD are the hippocampus, dorsolateral prefrontal cortex and anterior cingulate cortex. Later studies shifted towards comparing the neurological underpinnings of BPD to other disorders until a systematic and integrative review was published by Ruocco and Carcone (37), who discussed future directions including but not limited to gene x environment interactions, urging to explore novel applications of neuroscience findings to treatment research. Winsper et al. (38) showed through their systematic review some evidence for gene x environment interactions in childhood and adolescence as well. BPD patients with a risk of committing suicide are evidenced to heavily have dysfunction in the fronto-tepomral network, primarily involving reductions of grey and white matter volumes in the prefrontal cortex (PFC), anterior cingulate, and superior temporal gyrus (39), showing the critical role of maladaptive neuroplasticity of those specific brain regions on suicide. White matter pathology and alterations among BPD were also confirmed by later reviews (40, 41). Since then, most reviews confirm earlier findings and merely show more nuanced differences (42–49), but the main problem remains untouched.

To date, since the key understanding of suicidal thoughts and behaviors among BPD in response to reductions of grey and white matter volumes in the PFC and other regions, the physical environment has been an afterthought in BPD-related research. By the time no more relevant research for BPD in that regard was conducted, research on the impact of the environment (built and natural) on neuroplasticity has been growing exponentially, providing a plethora of neuroscience evidence on how the urban environment heavily influences such maladaptive plasticity outcomes. Even if treatment-induced brain plasticity is proven to effectively down-regulate neuronal activity within the insula and amygdala with the employment of prefrontal areas, orbitofrontal cortex, anterior cingulate cortex and dorsolateral prefrontal cortex along with enhancing functional connectivity between limbic and prefrontal regions (50), the realisation that heavy reliance on psychotherapy and drugs for a lifetime, to possibly counterbalance the fact that a large population is not responding to psychotherapy for BPD (51), alarms that the physical environment is the invisible and silent architect of BPD. The subsequent sections elaborately explain how those insights on neuroplasticity show that BPD patients are at high risk in urban environments, how natural environments and spatial complexity can foster adaptive neuroplasticity for BPD patients, and what the recommended calls to action are.

## 4 Physical environment factors causing similar maladaptive neuroplasticity outcomes found in BPD

The previous section provided an overview of research on brains with BPD, showing that specific brain regions explain the complexity of BPD. This section explores the physical environment variables associated with those brain regions and how the environment can become a source of positive adaptive or maladaptive neuroplasticity.

Regarding the amygdala, upbringing in rural regions showed higher right amygdala volumes compared to adolescents exclusively brought up in cities (52), while Kühn et al. (53) revealed later that the opposite of city living, forest coverage, and not even urban greenness, was associated with amygdala integrity. Nonetheless, several key studies can be relied on to prove that the urban environment can trigger emotional dysregulation in BPD at multiple levels through chronic exposure during adulthood. One of the earliest studies by Lederbogen et al. (54) showed that city living was associated with increased amygdala activity. A recent study compared two types of environments (natural and urban), showing that a 1-hour walk in urban and natural environments resulted in decreasing the amygdala activation after the walk in a forest, but it remains stable after the walk in an urban environment, urging urban planning to create more accessible green areas to adapt urban environments to benefit citizens’ mental health (55). Several EEG studies are found today to be consistent with the sophisticated insights provided by fMRI regarding the exposure to natural environments, urbanisation and mental disorders in urban communities (56), which can be of high relevance to the discourse in this article. Recently, Harris et al. (57) explained in their study that not only the absence of green space but also the presence of grey space (e.g., impervious surfaces as concrete, streets and rooftops) was linked with increased left amygdala-DMN connectivity, circuits implicated in affective processing, emotion regulation, and psychopathology. In other words, grey space increases the risk of emotion dysregulation as explained by the authors. Furthermore, greater amygdala activity is found in response to objects with sharp corners compared to curved ones (58–60). What is interesting, but also strikingly challenging, is that those studies were conducted on non-BPD patients. This urges us to consider the adverse effects of urban environments characterized by a lack of dense green space (forest-like coverage) and increased grey space (concrete and streets). Such conditions can drive emotional dysregulation in BPD patients, especially after acute daily exposure that becomes chronic. This is particularly concerning given that BPD patients not only exhibit a reduced amygdala size but also show excessive amygdala activation in response to stimuli, as previously explained. The 13% reduction in amygdala volume in BPD patients (31) can highly be expected to be triggered by city upbringing. Besides, environmental factors in the city can continuously trigger amygdala hyperactivity chronically, contributing to the hypervigilance, emotional dysregulation, and disturbed interpersonal relationships. BPD patients can experience engaging in higher diversity since it is proven to increase hippocampal volume (61), but that has to be cautiously explored with amygdala-dependent emotional dysregulation.

Hippocampal volume is also subject to atrophy in response to city upbringing where Kühn et al. (52) showed that rural upbringing was associated with higher volumes in the bilateral hippocampus of adolescents compared to those brought up in cities. Nonetheless, walkability in the environment is also associated with hippocampus volume. For instance, Cerin et al. (62) explained that each unit in walkability within 1 km radius residential buffer results in 0.038 to 0.043 cm^3^ greater right hippocampal volume. Cities are even rarely complex to navigate, while research shows that navigation training, with the effect of walking excluded, shows an increase in right hippocampus volume followed by a return to baseline, protecting the hippocampus from volume decline (63). Geospatial environmental complexity has been proven as well recently to have positive effects on various brain regions including the hippocampus (64). Walking itself is proven to increase hippocampal volume (65). Knowing that BPD patients show 11% decrease in hippocampus volume compared to health subjects (31), reliance on transportation, reduced walkability potential in cities, and low complex navigation can have dramatic adverse effects on hippocampus volume.

Regarding the cortical regions identified (premotor, prefrontal and anterior cingulate cortex), Dadvand et al. (66) showed that children with lifelong exposure to greenness were positively associated with grey matter volume in the left and right prefrontal cortex and in the left premotor cortex and with white matter volume in the right prefrontal region, in the left premotor region, and in both hemispheres. Furthermore, Kühn et al. (67) showed in their study on children’s upbringing (200m buffer) that there is a positive correlation between grey matter volume in various prefrontal clusters and green open space coverage (with sky views), but a negative association between within prefrontal clusters for tree cover density. Those studies indirectly provide very critical information on potential contribution of cities into the development of BPD where low exposure to greenness along with the existence of high-rise buildings and minimal setbacks inhibit skyviews and potentially lead to increased risk of BPD. Furthermore, Dzhambov et al. (68) show that the Normalised Difference Vegetation Index (NDVI) (500m buffer) is positively associated with average cortical thickness across both hemispheres among adults, more specifically in several gyri across the prefrontal cortex. Kühn et al. (69) show in their study that there is a negative association between urban fabric coverage and a positive association between urban green coverage and grey matter volume in perigenual/subgenual anterior cingulate cortex (p/sACC) among older adults (1 km buffer). Additionally, Baena-Extremera et al. (70) showed that people who exercise in green space exhibited increased thickness in the right anterior cingulate cortex (rACC). Collectively, those studies assert the importance of exposure to green space and sky views for the prevention of triggering BPD in people with genetic predisposition and preservation of a positive adaptive neuroplasticity across the course of life.

Last but most importantly, where grey and white matter volumes can drive BPD to the risk of suicide, some environmental variables are strongly evidenced to be associated with neuroplastic changes in both the grey and white matter volumes. Shang et al. (71) show that greater proximity to greenspace (100-300m buffer) was associated with larger total brain volumes, grey matter, and white matter, supporting the study on white matter presented by Dadvand et al. (66). In that regard, exposure to green spaces in cities within close proximity to the place of residence is very critical towards reducing suicidal risks among patients with high BPD severity.

## 5 Environment-BPD plasticity pathways, a need for collaborative treatment, and limitations

**Figure 1:**
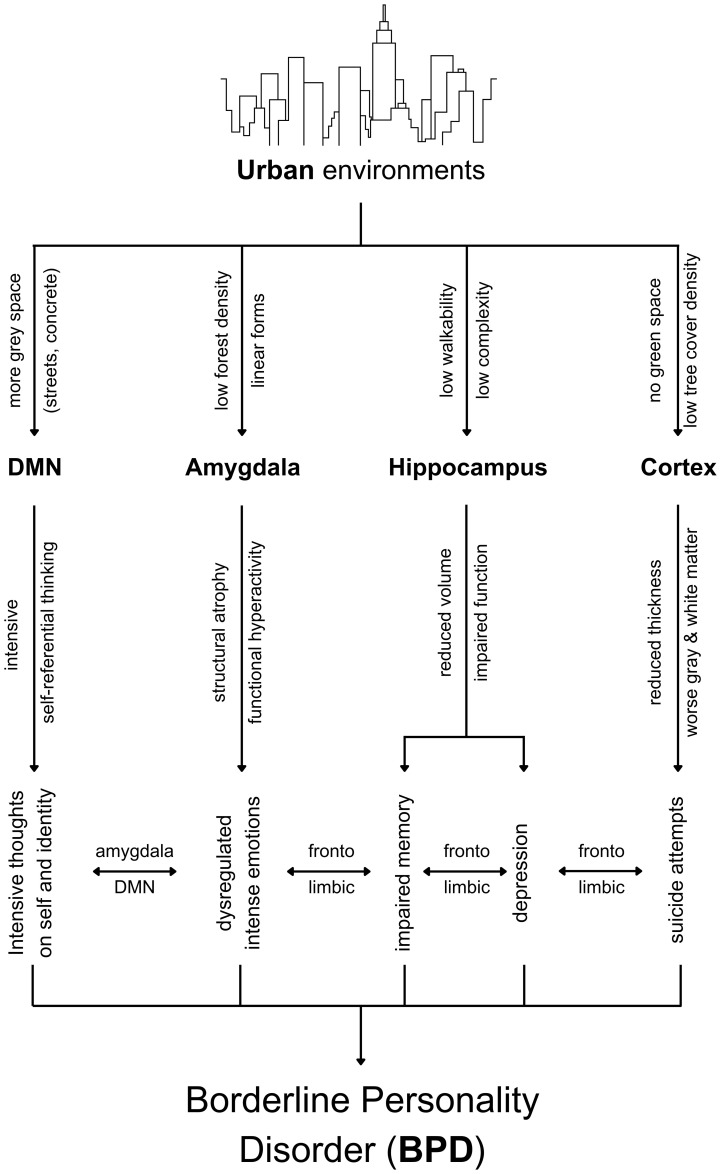
The urban environment-BPD neuroplasticity pathways.

Based on the earlier discussion, **Figure 1** illustrates the synthesis of the neuroplasticity dynamics linking urban living and BPD.

It is important to note that the complexity of BPD stems from the complexity of adverse effects resulting from hippocampal atrophy, amygdala atrophy and hyperactivity, reduced thickness in the prefrontal cortex, and worsened grey matter and white matter. Altogether, this article explains why BPD is given antidepressant medications and mood stabilizers (72) when the complexity of neuroplasticity processes is making BPD complex and that this complexity itself is driven by multiple variables in the physical environment, particularly urban environments for the reasons explained previously and illustrated in **Figure 1**.

This article strongly predicts that the physical environment is a significant triggering factor for BPD in patients with genetic predisposition and worsens the case during adulthood. Treatment should begin from childhood and upbringing to avoid triggering BPD and inhibit positively adaptive early development of the brain regions associated with BPD—amygdala, hippocampus and cortex—in response to the environment where those regions subsequently show dysfunction for a lifetime. Adulthood chronic exposure to cities worsens BPD symptoms and can lead to worsening grey and white matter that may highly contribute to suicide in BPD. It is not easy to begin treatment at the environmental level, but inevitably, the no-hope-for-treatment in BPD will forever persist if the environment walks in the opposite direction of treatment. For instance, if psychotherapy takes place on a weekly or biweekly basis while medical drugs overcome the complexly intertwined depression and anxiety symptoms, increasing dosage will only try to cope with the adverse neuroplasticity-based effects caused by the urban environment. BPD therapy, therefore, cannot take place without collaborative efforts from public health policymakers, the American Psychiatric Association, psychiatrists, and psychotherapists, on the one hand, to urban planners, urban designers, local governments setting building codes, architects, and even interior designers on the other hand, while BPD patients themselves should have more awareness about the physical environment impact through advocacy and education groups.

Knowing the dramatic adverse impacts of urban environmental living on BPD asserts protecting BPD from stigma and discrimination, which are very common among BPD (73–75), urging to face the main problem, which is how urban living is triggering BPD and driving it to a crisis. BPD patients and their families or caregivers often experience recurring crises with structural stigma specific to BPD remains pervasive in health systems reflected by factors embedded in institutional policies, cultural norms and practices, including BPD-related stigma and discrimination and dominance of a biomedically-driven approach to healthcare (76).

### 5.1 Collaborative BPD treatment

Public health has been urged for a few years to recognize BPD as a severe mental health disorder at all levels, highlighting a need for an evidence-based policy to address BPD (77), which this article not only strongly supports and strengths with neuroevidences but also advances the discourse with means for prevention and adaptive treatment through the discussed environment-BPD triggering interactions from genetic predisposition to behaviors after BPD is triggered. The shortage of BPD treatment providers across 22 countries (78) alarms an urge to shift perspective into environmental health through the environment-neuroplasticity dynamics to optimize current treatment plans with the current rations given. This article strongly supports the need for a more in-depth investigation of BPD care pathways (79), urging an investigation of the impact of environmental factors.

The American Psychiatric Association’s Diagnostic and Statistical Manual of Mental Disorders (DSM) is recommended to update the description of the factors influencing BPD to include physical environmental factors as well besides childhood adversity, abuse, and unstable family environments, which are all equally important but the equal important of the physical environment needs to be critically considered.

Psychiatrists are highly advised to take into consideration the chronic impact of the physical environment on BPD patients if they prescribe pharmacological medication for the complex symptoms accompanying the disorder. For instance, it was found that second-generation antipsychotics, anticonvulsants, and antidepressants were not able to consistently reduce the severity of BPD (9). The hypothesis presented in this article is that if medication will consistently overcome the adverse effects of the physical environment on the neuroplasticity of the amygdala (reduced volume and hyperactivity), hippocampus (atrophy) and cortex (reductions of grey and white matter volumes) combined then, it is doubtful that the BPD patient may have a sustainable long-term benefit from treatment without taking into account the synchronous impact of the physical environment.

Psychotherapists are more likely than psychiatrists to work with BPD patients more frequently. To date, dialectical behavior therapy (DBT) and schema therapy (ST) are the two most common psychotherapy approaches used by psychotherapists working with BPD patients, and both are effective to a great extent (80, 81). ST focuses on reorganizing the inner structure (82), while DBT provides a therapeutic response in the reduction of self-injurious behaviors, suicidal thoughts and attempts, the frequency of emergency care and hospitalisation in BPD (80), but as clear as the results indicate a ‘reduction’ of those symptoms, living in an urban environment that continues to worsen the grey and white matter as explained due to the lack of green spaces access and lack of surrounding tree cover density cannot wholly prevent those symptoms. Simple, BPD patients may see their therapists weekly or biweekly, but their exposure to their physical environment is chronic and is daily, which highly increases degenerative neuroplasticity and increases the risk of suicide. More than 75% of BPD patients have been reported to self-injure, while suicide rates are estimated to be as high as 10%, which is 50 times higher than in the general population (83, 84), urging psychotherapists to plan for access to green spaces more frequently as part of the psychotherapy plan using either DBT or ST because we argue in this opinion article that earlier suggestions by Thornton et al. (85) that DBT can improve neural plasticity will be negatively counterbalanced by the constant adverse impact of the physical environment.

While public health policymakers, psychiatrists and psychotherapists are encouraged to handle existing BPD crises, urban planners, urban designers, local governments setting building codes, architects and interior designers are equally called to take part by preventing design parameters from triggering BPD and worsening the symptoms for current BPD patients to an extent that can drive them to commit suicide.

Urban planners are strictly urged to be aware that their decisions have lifelong neuroplasticity outcomes not only for BPD patients but also the general public health, but BPD is more vulnerable, as explained in this paper. In that regard, urban planners must provide increased walkability opportunities within the 1 km buffer around the residential home address (62), suggested to be accompanied by complex navigation opportunities (64), in order to increase the likelihood of increasing the total hippocampus volume size in response to walking and the right hippocampus volume size in response to navigational-training and also walking in the environment. Designing urban environments with a diversity of zones and activities is also promising for increasing the hippocampus volume size as suggested by recent evidence indirectly (61), urging zoning and land use planning to take this into consideration (86). Urban planners also must consider the availability of green spaces within every 100-300m from each subject’s home address ad it was proven to be correlated with white and grey matter that, if worsened due to the absence of green spaces, can highly increase the risk of BPD committing suicide in response to the adverse neuroplasticity-dependent outcomes. Urban forest planning should highly take this into consideration (87).

Urban designers play a key role in the liminality of urban planning and architecture for public health, BPD prevention and treatment through the environment. In that regard, urban designers need to maximize tree cover density in cities and specifically within neighborhoods but with a balance of maximizing sky views through the arrangement of trees as well as through the provision of open public spaces and plazas. Nonetheless, less use of grey materials (streets, rooftops, etc.) is highly suggested to reduce amygdala hyperactivity. Those insights add new layers to the identified important urban design parameters mainly focused on thermal considerations (88), to the interest in optimizing green and grey urbanism (89), to exploring health outcomes dependent on concrete (90), and to showing more sides of promoting open public spaces in urban spaces (91).

Local governments working on building codes and setbacks must further strengthen the importance of setbacks (92–95) (distance from road and land boundary) in order to maximize sky views as this variable is very effective in the early development brain stages can upbringing with more sky views can highly trigger or prevent BPD development through the subsequent neuroplasticity processes determined by the environment.

Architects can promote public health, prevent BPD triggering and facilitate treatment of existing BPD patients in several ways. Firstly, building with skylights or atriums can be challenging, but it also promises to promote positive adaptive neuroplasticity. Skylight design configurations and daylight performance have been widely explored recently (96, 97) and similar atriums (98, 99). Secondly, buildings can become biophilic to different extents based on sunlight, color, gravity, fractals, curves, detail, water, plants, representations of nature, and organization and complexity (100). Valentine et al. (101) conducted a pilot study examining the impact of biophilic architectural design on neuroinflammation, suggesting that exposure to buildings with higher degrees of biophilia may result in decreased neuroinflammatory activity, which we take as evidence to explain the indirect relevance for BPD due to having increased plasma levels of oxidative stress and inflammatory markers in addition to reduced levels of the brain-derived neurotrophic factor (BDNF) (102). Thirdly, in order to increase the levels of BDNF, recent research shows that architectural environments promoting more physical activity through their designs can increase BDNF levels (103) and that the homeostasis facilitated by natural environments can optimize the BDNF increase (104). Therefore, how architects can prevent BPD and reduce its symptoms is critically important and needs to be done in multiple ways, as explained, to promote public health.

Interior spaces were recently shown to increase the likelihood of experiencing depression, anxiety and cognitive impairment compared to getting out of the house (105). As previous recommendations highlighted how to promote public health, prevent BDP triggering and overcome its symptoms, evidence can suggest ways at the interior level as well. Interior designers or individuals who design their homes themselves are highly advised to avoid the use of industrial concrete or grey materials that can lead to increased amygdala-DMN connectivity that leads to an increased risk of emotion dysregulation (57), which was explained earlier as highly critical for BPD subjects. This design characteristic should also be applicable to public spaces. On the contrary, the integration of real flowers or real flower images, compared to mosaic flowers, in interior spaces can provide automatic recovery effects after physiological stress through a deactivation in the right amygdala-hippocampus region (106), which provides additional insights for biophilic design (107, 108).

All of the above also applies to healthy humans, but the outcomes of lacking those environmental considerations may not be as risky as for BPD. Those approaches will save BPD patients, inhibit triggering BPD in genetically predisposed individuals, and improve the health of non-BPD individuals. BPD patients and the public with genetic predispositions are advised to increase time spent in natural environments such as forests and parks until the implementation of the previous suggestions commences. **Table 1** outlines the negative environmental influences, maladaptive neuroplasticity outcomes, and proposed improvement strategies.

**Table 1:** Negative environmental influences, adverse structural neuroplasticity outcomes, and proposed improvement strategies through the environment.

Negative environmental factor	Maladaptive neuroplasticity outcomes	Proposed improvement strategies
Low forest density	Reduced amygdala integrity	Urban planners need to provide public spaces with highly dense tree cover density that transcends the classification of urban greenness. This approach may counterbalance the severe amygdala atrophy in BPD patients.
	Amygdala hyperactivity	Urban planners are highly encouraged to provide green spaces and parks accessible from the home address, while architects are encouraged to design biophilic architecture, and interior designers should integrate natural plants or images of real flowers in interior spaces.
Gray spaces	Increased amygdala-DMN connectivity	Urban designers, architects, and interior designers must minimize or avoid using concrete and grey materials in streets, rooftops, facades, and industrial-style interiors.
Linear forms	Greater amygdala activation	Architects and interior designers should prioritize curved geometry and not ones with sharp corners.
Low walkability	Hippocampal atrophy	Urban planners should priorities increasing walkability units within each 1 km buffer around the home.
Low walkability (physical activity)	Reduced brain-derived neurotrophic factor (BDNF)	Urban planners are encouraged to provide more pedestrian pathways, while architects are highly encouraged to adopt the novel strategy of environmental affordance for physical activity through structural enrichment of the layout.
Low complexity	Hippocampal volume	Urban planners are encouraged to increase geospatial zip code-based environmental complexity to increase the likelihood of increasing right hippocampal volume.
Low tree cover density	Reduced cortical thickness	Urban planners and designers must prioritize increasing tree cover density using, for instance, the normalised difference vegetation index (NDVI) to ensure high greenness density within the 100 to 300 m buffer around the home addresses.
No sky views	Worsen white and gray matter	Architects need to provide skylights and atriums, urban designers should provide open public spaces and plazas, and local governments must strictly enforce setback building code regulations.

### 5.2 Limitations

Three main limitations arise at this point. Firstly, ignorance of the present evidence is only expected to increase the BPD behavioral risks. Secondly, the sole reliance on the therapy or environmental design approaches without collaborative efforts. Treatment of BPD and preventing its trigger has not only to be done through encouraging a healthy social environment but also exposure to a healthy physical environment from the beginning and throughout life. Last but not least, while most presented evidence is explained using brain scans, EEG headsets are feasible (109, 110), providing a wide range of options that can be used by clinicians, designers, BPD patients and healthy individuals with genetic predisposition to be able to track risk points and observe mental health improvements.

## 6 Conclusion

The impact of the physical environment on BPD need not be overlooked or taken positively for granted anymore as urban living appears through neuroscience evidence to be directly in relationship with BPD. Key urban living variables are proven in this paper to cause the adverse neuroplasticity outcomes for the amygdala, hippocampus and cortex that together form the complexity of BPD and make it untreatable not only due to its complexity but because the physical environment does not stop worsening the crisis. With the needed actions exemplified in this article, urgent collaborative efforts are critically needed by therapists and environmental decision-makers alike where the hope for borderline personality disorder lies at the liminality of their effort. This article advocates that borderline personality disorder's black-and-white patterns should not be stigmatised. We need to shift our perspective towards the grey and linear city before we expect borderline personality disorder to think and behave on a grey spectrum.
